# Sun Exposure Guidelines and Serum Vitamin D Status in Denmark: The StatusD Study

**DOI:** 10.3390/nu8050266

**Published:** 2016-05-05

**Authors:** Louise Hansen, Anne Tjønneland, Brian Køster, Christine Brot, Rikke Andersen, Marika Lundqvist, Jane Christensen, Anja Olsen

**Affiliations:** 1 Unit of Diet, Genes and Environment, Danish Cancer Society Research Center, Copenhagen DK-2100, Denmark; annet@cancer.dk (A.T.); anja@cancer.dk (A.O.); 2 Department of Prevention and Information, Danish Cancer Society, Copenhagen DK-2100, Denmark; brk@cancer.dk; 3 Danish Health Authority, Health Promotion, Copenhagen DK-2300, Denmark; chb@sst.DK; 4 Technical University of Denmark, National Food Institute, Division for Diet, Disease Prevention and Toxicology, Research Group for Risk-Benefit, Søborg DK-2860, Denmark; rian@food.dtu.dk; 5 Statens Serum Institute, Department of Congenital Disorders, Danish Centre for Neonatal Screening, Copenhagen DK-2300, Denmark; mlq@ssi.dk; 6 Unit of Statistics, Bioinformatics and Registry, Danish Cancer Society Research Center, Copenhagen DK-2100, Denmark; jane@cancer.dk

**Keywords:** skin cancer, StatusD, sun exposure guidelines, vitamin D

## Abstract

Little is known on how vitamin D status is affected by adherence to UVB-limiting sun exposure guidelines. Our aim was to investigate the relationship between adherence to the Danish sun exposure guidelines and vitamin D status. In total, 3194 Danes (2625 adults, 569 children) were recruited among the general population, and more than 92% had blood samples taken both autumn and spring. Using linear regression, we associated serum vitamin D concentrations to questionnaire responses on: seeking shade, wearing a sunhat, wearing protective clothing or using sunscreen. The odds ratio (OR) of either low (<25 or 50 nmol/L) or adequate/high (≥50 nmol/L) vitamin D status was examined using logistic regression. For adults, those who always sought shade or wore protective clothing compared to those who did not had lower levels of vitamin D (autumn concentrations for shade: 7.2 nmol/L lower (−11.0–−3.6 nmol/L); for protective clothing: 9.9 nmol/L lower (−13.6–−6.2 nmol/L). Adherence to all four guidelines was also associated with lower vitamin D concentrations (autumn: 9.7 nmol/L lower (−14.3–−5.1 nmol/L). Use of sunscreen was associated with adequate vitamin D status, as those who always sought shade compared to those who did not had an OR (95% CI) of 1.68 (1.25–2.35) of having ≥50 nmol/L during both spring and autumn. No associations were found with wearing a sunhat, and there were no clear associations for children. In conclusion, adherence to the sun exposure guidelines on shade and protective clothing was associated with lower vitamin D status among Danish adults, but not children.

## 1. Introduction

Vitamin D production is highly dependent on solar exposure, and it is estimated that between 80% and 90% of our vitamin D status stems from UVB-induced production [1]. However, prolonged exposure to UVB radiation is also the major risk factor for both non-melanoma skin cancer and malignant melanoma [2]. During 2009–2013, more than 2000 new cases of malignant melanoma were diagnosed in Denmark, and more than 250 deaths were registered annually [3]. The Danish Sun Safety campaign entitled “Reduce your sun exposure between 12 and 3 p.m.” was launched in 2007 with the goal of reducing skin cancer incidence. The campaign includes four guidelines that focus on seeking shade, wearing a sunhat, wearing protective clothing and using sunscreen. These guidelines aim to limit Danes’ sun exposure during the time of day with the highest UV-index and should be applied whenever the UV-index is above three [4,5]. Sun exposure guidelines and protection programs also exist in several other countries, including the UK [6], Australia [7] and the US [8], and the World Health Organization (WHO) also advocates healthy sun behavior [9].

Insufficient vitamin D status may be related to several undesirable health consequences with regard to bone and skeletal development [10], and numerous studies have also related low levels of vitamin D to several chronic diseases, including cancer, heart disease, diabetes, and multiple sclerosis [11,12,13]. However, the literature is inconsistent on the association between vitamin D and incidence, prognosis and mortality of these chronic diseases. Some of the studies have even indicated that the association may be U-shaped, meaning that not only is there an increased risk among those with very low levels of vitamin D, but also among those with very high levels [14,15,16].

It is, however, well-known that vitamin D plays an important role during growth, and that a sub-optimal vitamin D status throughout childhood may increase the risk for later osteoporosis development [17]. For this reason, concern has been raised regarding negative impacts of the UVB exposure-limiting attempts on vitamin D status, since vitamin D production is directly related to sunlight exposure. Furthermore, in Denmark, many children’s day cares have sun exposure guidelines to limit harmful UVB exposure, and there have been concerns that this may negatively impact the vitamin D status of young Danish children.

It is therefore a delicate balancing act to ensure optimal vitamin D synthesis for bone growth as well as other vitamin D demanding biological functions while limiting the risk for particularly malignant melanoma. Sufficient vitamin D concentrations are produced in the summer even by short outdoor exposure times [18], but complete adherence to the sun exposure guidelines could theoretically lead to lower vitamin D synthesis caused by lower sun exposure. Little is known on how adherence to the guidelines is associated with vitamin D status, and our aim was to investigate the relationship between adherence to the Danish sun exposure guidelines and vitamin D status.

## 2. Methods

### 2.1. Study Description

The StatusD study was established in 2012–2014, and participants were randomly recruited from the general population in three major regions in Denmark (Copenhagen Central area (63%), Odense (19%), and Kolding (18%)) using the Danish Civil Registration System [19]. The study was approved by the Regional Ethics Committee in Copenhagen (H-4-2012-068), and all participants gave written consent at the onset of the study. Inclusion criteria to the study were: age between 2 and 69 years and not included in any of the groups who are recommended to take vitamin D supplements by the Danish Health Authority [20]. This covers persons: (1) with dark skin; (2) who wear protective clothing or covering during the summer (e.g., due to religious reasons); (3) who are rarely outside or actively avoid sunlight; (4) who live in a nursing home; or (5) who are at increased risk of developing osteoporosis. Vitamin D supplements are also recommended during pregnancy, and pregnant women could therefore not be enrolled in the study; however, they were still encouraged to have their second blood sample drawn if they became pregnant between the two samplings.

**Figure 1:**
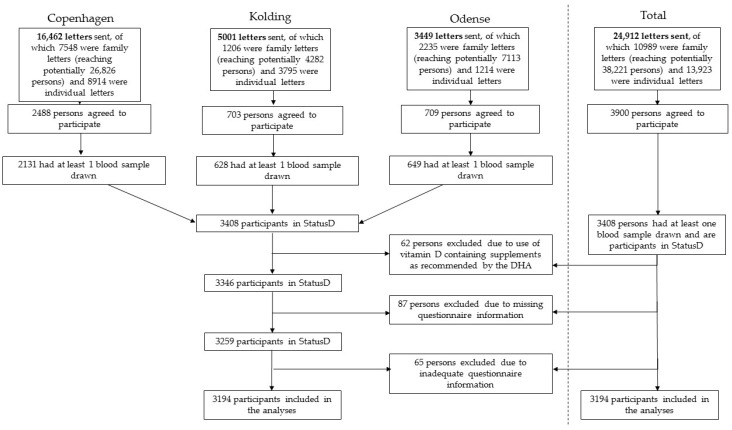
StatusD participant recruitment. DHA: Danish Health Authority.

Participants were invited by letter to participate in the study, and we contacted individuals and families separately. Families were defined as consisting of at least one person above 18 years and one child below 18 years; however, there was no upper limit on number of family members. Thus, a letter that was sent to a family could potentially reach and invite all family members living at the same address (*i.e.*, between 2 and 10 persons per letter). It is therefore difficult to accurately determine a participation rate for StatusD, as we do not know the exact number of potential participants. As shown in Figure 1, we sent a total of 24,912 letters, and 3900 persons agreed to participate.

Of the 3900 persons who signed up to participate, 3408 persons came to the study center and had at least one blood sample drawn; more than 92% had blood samples drawn at both autumn and spring blood collections. Use of supplements containing vitamin D was not cause for exclusion for the majority of the StatusD participants. However, of the 3408 persons in StatusD, 62 persons were excluded after indicating on their questionnaire that they consumed vitamin D-containing supplements due to one of the abovementioned criteria set forth by the Danish Health Authority. Of these 62 persons, 3 answered that they had dark skin, 4 wore protective clothing or covering during the summer, 39 were rarely outside or actively avoided sunlight, 14 were at increased risk of developing osteoporosis, and 2 were pregnant at time of inclusion. A further 87 persons were excluded, as they did not complete an autumn questionnaire, where the questions on sun behavior were included. The analyses in this paper are limited to sun behavior on a day off, as we believe this to be a better reflection of a person’s true sun behavior than on workdays. Therefore, a further 65 persons were excluded as they responded that they were not outside at any time on a day off during the week. The total study population for this paper is therefore 3194 persons, encompassing 569 children (<18 years) and 2625 adults (≥18 years).

### 2.2. Measurement of Vitamin D Status

Serum samples were analyzed for 25-hydroxyvitamin D (25(OH)D, hence denoted vitamin D) concentrations as the sum of 25(OH)D3 and 25(OH)D2) as previously described [21] at Statens Serum Institute. This was done by liquid chromatography-tandem mass spectrometry (LC-MSMS, Copenhagen, Denmark) in positive mode using the “MSMS vitamin D” kit from Perkin Elmer (Waltham, MA, USA). Statens Serum Institute participates in DEQAS (Vitamin D External Quality Assessment program).

The samples were run in a randomized order, but spring and autumn samples for each person were analyzed in the same run. All samples were measured by single determination. The precision study included 5 runs conducted by 1 operator. Within-run precision was calculated from 3 replicates for each sample, and between-run precision was calculated from the average values for each run; the total coefficient of variation was below 15%.

### 2.3. Questionnaire Information and Exposure Definition

Participants were recruited either during the spring or during the autumn and filled in an electronic questionnaire, encompassing questions on background (education, employment, housing type, and ethnicity), lifestyle, diet, use of dietary supplements and sun behavior. The questionnaire we have used is not validated in its entirety, but is a combination of questions from three questionnaires, including one used to collect data in the Diet, Cancer and Health cohort (dietary and lifestyle questions) [22] and one used to collect data on adherence to the sun exposure guidelines [23]. The questions on use of supplements and ethnicity come from a third questionnaire used in the VitmaD project [24].

Participants filled in a questionnaire at both blood samplings, meaning that an autumn blood sample can be combined with an autumn questionnaire and *vice versa* for the spring. As the questionnaire was completed electronically, there was no possibility to skip a question. The questionnaire for the children was either filled in by a parent, in collaboration with the child, or, for the older children, by themselves. Children aged 15–18 filled in an adult questionnaire, which had slightly more questions (on, e.g., smoking and alcohol) than the children’s questionnaire. The children were specifically asked whether their day care/kindergarten/after school club (hence termed “institution”) had guidelines regarding sun exposure. Height and weight were measured at the study center by trained personnel and used to calculate BMI (kg/m^2^) for adults. BMI z-scores were calculated for children between 2 and 17 years using z-score data tables [25] and cut-offs for overweight were used according to the WHO guidelines [26].

In the autumn questionnaire, participants were asked about their sun exposure behavior on a sunny day off between 12 and 3 p.m. There were four questions asking them whether they: (1) Seek shade; (2) Wear a sunhat; (3) Wear clothing that covers body, upper arms and thighs; and (4) Use sunscreen. For each question there was 4 possible answers: “Always”, “Often”, “Occasionally”, and “No”. Furthermore, they were asked how many days off during a week that they were outside for shorter or longer periods of time. Finally, the participants were asked for how many minutes or hours they were outside on a typical day off in the time periods 9 a.m.–12 p.m., 12–3 p.m. and 3–6 p.m. On the question of sun exposure guidelines in the institution, possible answers were: “Yes”, “No”, and “Unsure”.

We looked at spring and autumn vitamin D status separately in combination with each of the four sun exposure guidelines as well as in a combined index with yes/no combinations for each of the four guidelines (with 5 categories ranging from 0: fulfilled 0 of the sun exposure guidelines to 4: fulfilled 4 guidelines). The answers “always” and “often” were combined to “yes”, while “no” included “occasionally” and “no”. Furthermore, we investigated the odds ratio (OR) of having a low (≤25 or ≤50 nmol/L) or an adequate/high vitamin D (≥50 nmol/L) status in relation to the sun exposure guidelines. Vitamin D below 25 nmol/L is termed vitamin D deficiency, whereas concentrations between 25 and 50 nmol/L are termed insufficient [27]. For these analyses, four levels were assessed, ranging from undesirable vitamin D levels to desirable vitamin D levels: (1) those having a vitamin D level below 25 nmol/L during either spring or autumn (level 1); (2) those having a vitamin D level below 50 nmol/L during both spring and autumn (level 2); (3) those having a vitamin D level equal to or above 50 nmol/L during either spring or autumn (level 3); and (4) those having a vitamin D level equal to or above 50 nmol/L during both spring and autumn (level 4). We would have liked to include a level with those who have a vitamin D concentration below 25 nmol/L during both spring and autumn, but only 20 persons (0.6% of StatusD) fulfilled this.

We also assessed the combined effect of adherence to the sun exposure guidelines and total sun exposure during a day off between 9 a.m. and 6 p.m. Total sun exposure was calculated by multiplying the amount of time (in hours) spent outside in the three time intervals, 9 a.m.–12 p.m., 12–3 p.m. and 3–6 p.m., with the number of days off and weighted according to the amount of expected UV-radiation in that time interval. The UV index peaks between 12 and 3 p.m., where approximately half of the total UV radiation for the entire day is found [28]. This therefore equaled to 50% for both 9 a.m.–12 p.m. and 3–6 p.m., meaning that, e.g., 1 h spent outside between 9 a.m. and 12 p.m. only counted as 0.5 h, while 1 h between 12 and 3 p.m. counted as 1 h.

### 2.4. Statistical Analyses

All analyses were performed in SAS Enterprise ver 5.1 (SAS Institute Inc., Cary, NC, USA). Vitamin D concentrations were tested for normal distribution using a QQ plot and assessed visually, and no deviation from linearity was seen. Sex, age at first blood sampling and use of vitamin D supplements were tested in a linear regression model using the PROC GLM procedure and found to be significantly different for all three variables (*p* < 0.001) and therefore included as covariates in all analyses. Analyses correlating vitamin D concentrations during either spring or autumn to adherence to the sun exposure guidelines (also in the children’s institution) as well as the interaction models were performed with linear regression using the PROC GLM procedure. The analyses on OR of having a low or adequate/high vitamin D status in relation to sun exposure guidelines were performed with logistic regression using the PROC GENMOD procedure.

All analyses were conducted separately for children (<18 years) and adults (≥18 years). Persons were designated as vitamin D users if they consumed any supplement containing vitamin D as indicated on their spring questionnaire; this questionnaire was chosen, as we assessed this to be the best reflection of supplement use, since supplements are more often taken during autumn and winter. There were 22 women (1%) who became pregnant between the two blood samplings, and sensitivity analyses were performed to assess the possible impact of this. No changes in the results were seen, and they were included in the final data analyses.

## 3. Results

Children in StatusD ranged from 2 to 18 years, and 63% were in the age group 6–15 years. There were almost equal numbers of boys and girls. Many of the children reported being physically active during their leisure time (87%), and 37% reported taking supplements containing vitamin D. For adults, there was an almost even distribution in the five age groups, with slightly fewer in the youngest age group (18–29; 14%) and slightly more in the age group 40–49 years (26%). More women than men participated (59% *vs.* 41%), and the majority of the adult population were non-smokers (58%). Similar to the children, the majority were physically active (75%) and 42% reported taking supplements containing vitamin D. Almost half (46%) were in the normal BMI range of 18.5–25, while 38% were overweight (BMI 25–30) and 15% were obese (BMI 30+) (Table 1).

Table 1 also shows the distribution for both children and adults with regard to the sun exposure guidelines. The sun exposure guideline that was adhered to the least concerns the use of sunhat, where 69% of the children and 70% of the adults replied that they never wore a sunhat. However, most children replied that they always or often wore sunscreen (30% and 36%, total 66%), and almost half of the adults wore sunscreen (18% and 30%, total 48%).

Median (5%–95%) concentrations of vitamin D among children were 67.4 nmol/L (40.3–99.0 nmol/L) and 44.0 nmol/L (18.5–84.2 nmol/L) in the autumn and spring, respectively. When limited to those without use of vitamin D supplements, the concentrations were slightly lower (median (5%–95%) in autumn: 65.9 nmol/L (38.6–100.5 nmol/L); in spring: 36.3 nmol/L (16.7–71.3 nmol/L)). Among adults, median (5%–95%) concentrations of vitamin D were 72.2 nmol/L (38.8–114.1 nmol/L) and 50.0 nmol/L (19.0–98.2 nmol/L) in the autumn and spring, respectively. When limited to those without use of vitamin D supplements, the concentrations were slightly lower (median (5%–95%) in autumn: 68.4 nmol/L (36.1–111.6 nmol/L); in spring: 40.0 nmol/L (16.5–84.6 nmol/L)) (results not shown in table). These values are in Table 1 combined to illustrate the number of children and adults, respectively, which have concentrations that fall below the recommended level of 50 nmol/L. Only 14% of children and 13% of adults have vitamin D concentrations below 50 nmol/L during both spring and autumn (level 2); most have concentrations below 50% during spring that then rise above 50 nmol/L during autumn. However, 13% of children and 11% of adults have concentrations below 25 nmol/L during either spring or autumn (level 2). Almost half (47%) of adults have concentrations above 50 nmol/L during both spring and autumn (level 4).

Table 2 shows the relationship between adherence to the sun exposure guidelines or the combined index and vitamin D concentrations. For children, no significant associations with vitamin D concentrations were seen during spring or autumn for any of the guidelines or the index. For adults, significantly lower vitamin D concentrations were seen for the sun exposure guidelines on seeking shade and for wearing protective clothing as well as in relation to adherence to the combined index. Those who always sought shade had 9.9 nmol/L lower vitamin D concentrations in autumn (95% CI: −12.5–−6.2)); the value was slightly less in spring (−7.2 nmol/L (−11.0–−3.6 nmol/L)). Those who often or occasionally sought shade also had significantly lower vitamin D concentrations (however only significantly for those who often sought shade in autumn). The same pattern was seen for those who wore protective clothing, with the greatest difference seen in the autumn concentrations and for those who always followed the guideline (−9.9 nmol/L (−13.6–−6.2 nmol/L)), but those who often or occasionally followed the guidelines also had significantly lower concentrations of vitamin D. For the index, increasingly lower vitamin D concentrations were seen among those who followed one or more guidelines with the clearest pattern seen in autumn and for those who followed all four guidelines (−9.7 nmol/L (−14.3–−5.1 nmol/L)).

We also assessed how adherence to the sun exposure guidelines was associated with the OR of having low vitamin D status (level 1: <25 nmol/L either spring or autumn; level 2: <50 nmol/L both spring and autumn) (Table 3). For children, there were very few significant results. Always adhering to the sun exposure guidelines concerning shade, sunhat or wearing protective clothing was associated with a higher OR of belonging to level 1. Likewise, those who always sought shade had a higher OR of belonging to level 2. For adults, there was a higher OR of belonging to levels 1 and 2 among those who always or often followed the guideline concerning shade. The highest OR was seen for those who often sought shade; they had an OR (95% confidence interval (CI)) of 2.17 (1.50–5.15) of having vitamin D status below 50 nmol/L during both spring and autumn. The same tendency was seen for the guideline on wearing protective clothing, where all categories were associated with a higher OR of having lower vitamin D status. The highest OR was seen for those who always wore protective clothing, as they had an OR (95% CI) of 3.19 (1.98–5.16) of having vitamin D concentrations below 25 nmol/L during either spring or autumn.

**Figure 2:**
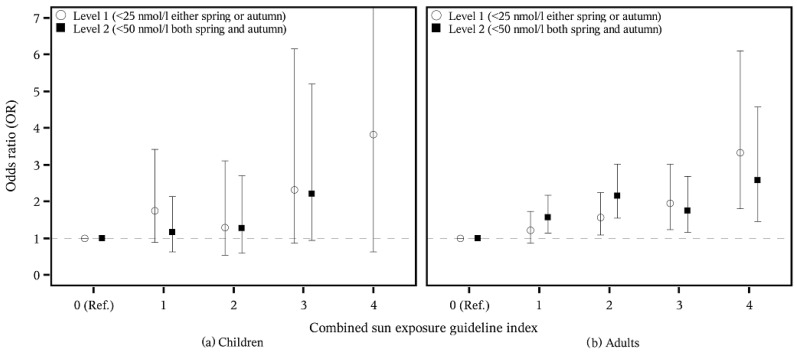
Adherence to the combined sun exposure guideline index and odds ratio (OR) and 95% CI of having low vitamin D (levels 1 and 2) among children, *n* = 569 (**a**) and adults, *n* = 2625 (**b**).

For the combined index, a pattern of having higher OR of low vitamin D status in relation to greater adherence to the index was seen for adults but not for children (Figure 2). Those who adhered to two or more guidelines had a higher OR of having vitamin D concentrations below 25 nmol/L (level 1), and those who adhered to all four guidelines had the highest OR: 3.32 (95% CI: 1.81–6.10). The same was true for level 2, where also those who only followed one guideline had a higher OR of having vitamin D concentrations below 50 nmol/L during both autumn and spring. The highest OR was again seen for those who followed all four guidelines (OR (95% CI): 2.58 (1.46–2.57)).

Table 4 shows how adherence to the sun exposure guidelines was associated with the OR of having adequate or high vitamin D status (level 3: ≥50 nmol/L either spring or autumn; level 4: ≥50 nmol/L both spring and autumn). For children, almost no significant results were seen; only those who always adhered to the guideline concerning shade had a lower OR of belonging to level 3. For adults, there was a consistently lower OR of belonging to both level 3 and 4 among those who always, often or occasionally followed the guideline concerning clothing. The lowest OR was seen for those who always followed the guideline (OR (95% CI) for level 3: 0.32 (0.21–0.50)). A lower OR was also seen concerning the guideline on shade. On the contrary, those who often or always used sunscreen had a higher OR of belonging to level 4 (OR (95% CI) for always: 1.68 (1.25–2.25)).

**Figure 3:**
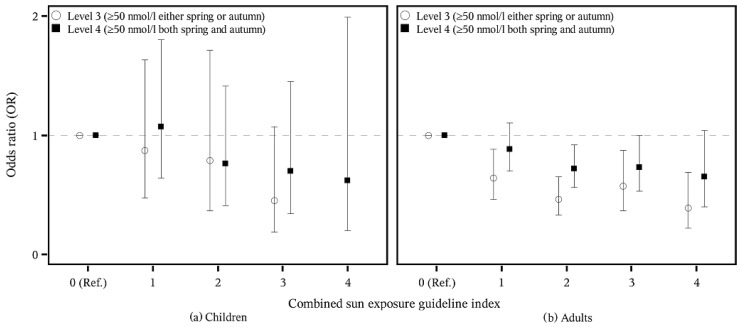
Adherence to the combined sun exposure guideline index and odds ratio (OR and 95% CI) of having adequate/high vitamin D (levels 3 and 4) among children, *n* = 569 (**a**) and adults, *n* = 2625 (**b**).

Lower OR of having adequate or high vitamin D status during autumn and spring (level 4) in relation to adherence to the combined index was seen among adults only for those who adhered to two guidelines with an OR (95% CI) of 0.72 (0.56–0.92) but with a tendency towards lower status for all index categories (Figure 3). However, those who adhered to 1–4 guidelines all had a lower OR of belonging to level 3 and therefore of having adequate vitamin D concentrations. The lowest OR was seen for those who adhered to all four guidelines (OR (95% CI): 0.39 (0.22–0.69)). No significant associations were seen for children.

Children between 2 and 15 years were asked whether their institution had guidelines regarding sun protection. Out of 440 children, 203 (46%) replied yes, 60 (14%) replied no, while 177 (40%) replied that they were unsure. There were no significant differences in vitamin D status, spring or autumn, for those with sun exposure guidelines in their institution compared to those without (Table 5). The same was true for those that were unsure. We were unable to perform analyses on the 2–6 year olds separately, as only one person answered that there were no sun exposure guidelines in the institution, and only six were unsure (compared to 78 who answered yes).

Adjustment for the potential confounders physical activity and BMI (kg/m^2^) (BMI z-scores for children), as well as smoking for adults only, was evaluated, but the changes were negligible, and the results are not shown. Analyses combining exposure time and sun exposure guidelines are shown in Table S1. Significant associations were only seen for children who adhered to the sun exposure guideline concerning shade.

## 4. Discussion

In this study on vitamin D among 3194 Danes recruited from the general Danish population, seeking shade and wearing protective clothing was significantly associated with lower vitamin D status in adults but not in children. Wearing a sunhat or using sunscreen was not significantly associated with lower vitamin D status among adults or children. Increasingly lower concentrations of vitamin D were found for adults who adhered to one or more of the sun exposure guidelines, and the lowest concentrations were seen for those who adhered to all four guidelines. The OR of having low vitamin D status during spring and/or autumn (assessed both at below 25 nmol/L and 50 nmol/L) was higher for those who always or often sought shade or wore protective clothing among adults. Conversely, the OR of having vitamin D concentrations above 50 nmol/L was lower for those who adhered to the same guidelines on shade and clothing. However, there was a higher OR of having vitamin D concentrations above 50 nmol/L for those who always or often wore sunscreen. No association between having sun exposure guidelines in children’s institutions and vitamin D concentrations was found.

Our results are consistent with a study performed in an American population where an association between sun protective behaviors and lower vitamin D concentrations was found. Here it was likewise seen that staying in the shade or wearing long sleeves was associated with lower vitamin D concentrations. Wearing a sunhat was not associated with lower vitamin D concentrations [29]. We also did not find that use of sunhat was associated with lower vitamin D concentrations. It is plausible that use of sunhat would not lead to lower vitamin D status, as a sunhat only covers a very small part of the body and thus only limits sun exposure slightly. However, it should be noted that 69% and 70% of children and adults, respectively, reported that they do not wear a sunhat when sun exposed.

There has been concern that frequent sunscreen use could lead to lower vitamin D synthesis in the skin, as this has been seen in strictly controlled situations [30]. However, we found that adults who used sunscreen had a higher OR of having vitamin D concentrations above 50 nmol/L during both autumn and spring. Sunscreens are often applied before intentional sun exposure, and it is therefore likely that the observed association is primarily a reflection of prolonged sun exposure [31]. It could also be that the sunscreen was applied incorrectly or irregularly or with a low SPF.

In this study, we have repeated measures for 92% of our study participants, including both a spring and an autumn measurement, which is a considerable strength. While no vitamin D synthesis takes place between October to March in Denmark, the spring level is still a reflection of the synthesis that occurs during the summer months, and the two measures in combination give a much clearer picture of seasonal variation than a single measurement. Furthermore, vitamin D measurements were performed using LC-MS, which is considered to be the most reliable laboratory method for analyzing vitamin D [32]. Validity and comparability of vitamin D measurements is an important factor to consider when comparing studies on vitamin D [33]. Finally, persons were recruited from the general population, and we included both children and adults.

Our study also had some limitations. The answers to the sun exposure guidelines were self-reported, and we only have one questionnaire that captured these for each person. Validation studies have shown that exposure to UV radiation assessed by retrospective questionnaires is only accurate for short period [34] but cannot be inferred directly between questionnaire and measured radiation for longer periods [35]. Thus, coincidence between, e.g., seeking shade and low exposure may have influenced the results. Furthermore, we did not collect information on chronic illnesses. Some chronic illnesses (e.g., kidney disease) may lead to lower vitamin D concentrations [36], thereby potentially affecting the results, and it would have been preferable to have this information to adjust accordingly for it.

In general, findings for adults and children were in the same direction, though more often statistically significant among adults. The smaller number of participants and thereby lower statistical strength may be part of the explanation for the non-significant estimates seen for children. Furthermore, the overall participation rate was quite low. When comparing the StatusD population to the general Danish population, our study population did seem to be slightly more health conscious: less obese, fewer smokers, more physically active (comparisons not shown). Although it is not clear how these factors could impact the associations studied, they may impact the external validity of the results.

Our findings are difficult to interpret in a public health context; the observational nature of our study does not allow conclusions on how changes in sun habits would affect serum vitamin D concentrations and furthermore, it is an ongoing discussion to what degree higher vitamin D concentrations are causally related to improved public health. The present study raises the observation that sun exposure guidelines may have lower population vitamin D status as a consequence. Further studies are needed to answer whether the guidelines should be different in order to optimally balance prevention of skin cancer with sufficient vitamin D status.

## 5. Conclusions

In the StatusD study, seeking shade or wearing protective clothing was associated with lower vitamin D status among Danish adults, whereas use of sunscreen and sunhat was not. Those who always or often sought shade or wore protective clothing also had a lower OR of having vitamin D concentrations above 50 nmol/L.

## Figures and Tables

**Table 1:** Distribution of selected variables among StatusD participants ^a^.

	Children (*n* = 569)	Adults (*n* = 2625)
**Age**		
≥2–<6	91 (16%)	-
≥6–<15	360 (63%)	-
≥15–<18	118 (21%)	-
≥18–<29	-	367 (14%)
≥30–<39	-	508 (19%)
≥40–<49	-	681 (26%)
≥50–<59	-	595 (21%)
≥60	-	514 (20%)
**Sex**		
Men	281 (49%)	1063 (41%)
Women	288 (51%)	1562 (59%)
**Smoking**		
Never	-	1528 (58%)
Former/occasional	-	712 (27%)
Current	-	385 (15%)
**Physical activity**		
Yes	493 (87%)	1978 (75%)
No	76 (13%)	647 (25%)
**Use of supplements containing vitamin D**		
Yes	213 (37%)	1092 (42%)
No	356 (63%)	1533 (58%)
BMI		
<18.5	-	32 (1%)
≥18.5–≤25	-	1220 (46%)
>25–≤30	-	986 (38%)
>30	-	387 (15%)
**Adherence to sun exposure guideline on shade**		
Always	15 (3%)	217 (8%)
Often	117 (21%)	832 (32%)
Occasionally	327 (57%)	1172 (45%)
No	110 (19%)	404 (15%)
**Adherence to sun exposure guideline on sunhat**		
Always	16 (3%)	126 (5%)
Often	50 (9%)	243 (9%)
Occasionally	110 (19%)	425 (16%)
No	393 (69%)	1831 (70%)
**Adherence to sun exposure guideline on protective clothing**		
Always	49 (9%)	164 (6%)
Often	143 (25%)	568 (22%)
Occasionally	216 (38%)	898 (34%)
No	161 (28%)	995 (38%)
**Adherence to sun exposure guideline on sunscreen**		
Always	168 (30%)	478 (18%)
Often	207 (36%)	790 (30%)
Occasionally	142 (25%)	813 (31%)
No	52 (9%)	544 (21%)
**Adherence to combined sun exposure guideline index**		
0 guideline	122 (21%)	703 (27%)
1 guideline	236 (42%)	924 (35%)
2 guidelines	122 (21%)	604 (23%)
3 guidelines	71 (13%)	290 (11%)
4 guidelines	18 (3%)	104 (4%)
**Vitamin D groupings based on status ^b^**		
Level 1: <25 nmol/L either spring or autumn	75 (13%)	294 (11%)
Level 2: <50 nmol/L both spring and autumn	81 (14%)	350 (13%)
Level 3: ≥50 nmol/L either spring or autumn	488 (86%)	2275 (87%)
Level 4: ≥50 nmol/L both spring and autumn	204 (36%)	1235 (47%)

^a^ Number (*n*) and % out of total children (*n* = 569) or total adults (*n* = 2625); ^b^ The levels are not mutually exclusive and do therefore not combine to 100%. The numbers show how many children or adults (%) are included in each individual level.

**Table 2:** Difference in vitamin D concentrations (nmol/L) according to adherence to the sun exposure guidelines and the combined index when the sun is shining between 12 and 3 p.m. in children (*n* = 569) and adults (*n* = 2625) in StatusD ^a^.

	Children		Adults	
	Spring (β (95% CI))	Autumn (β (95% CI))	Spring (β (95% CI))	Autumn (β (95% CI))
**Shade**				
Always	−9.7 (−20.6–1.3)	−13.5 (−23.3–3.6)	−7.2 (−11.0–−3.6)	−9.9 (−12.5–−6.2)
Often	−0.3 (−5.1–4.4)	−3.2 (−7.8–1.5)	−7.2 (−9.9–−4.6)	−7.6 (−10.3–−5.0)
Occasionally	0.6 (−3.3–4.5)	1.6 (−2.3–5.4)	−3.7 (−6.2–−1.3)	−2.3 (−4.8–0.2)
No	0	0	0	0
**Sunhat**				
Always	3.5 (−6.4–13.4)	3.9 (−5.5–13.3)	−2.9 (−6.9–1.2)	−2.6 (−6.7–1.5)
Often	−1.3 (−7.1–4.4)	−1.9 (−7.6–3.8)	−1.3 (−4.3–1.8)	−1.0 (−4.0–2.1)
Occasionally	2.5 (−1.8–4.5)	−0.7 (−5.0–3.5)	0.3 (−2.1–2.7)	0.7 (−1.7–3.1)
No	0	0	0	0
**Clothing**				
Always	−4.8 (−10.8–1.1)	−4.4 (−10.3–1.5)	−9.1 (−12.7–−5.4)	−9.9 (−13.6–−6.2)
Often	−3.7 (−8.1–0.7)	−3.3 (−7.6–3.8)	−6.8 (−9.1–−4.5)	−8.1 (−10.4–−5.8)
Occasionally	−1.0 (−4.7–2.8)	−0.7 (−5.0–3.5)	−4.5 (−6.5–−2.5)	−4.7 (−6.7–−2.7)
No	0	0	0	0
**Sunscreen**				
Always	−2.0 (−4.0–8.1)	1.9 (−4.1–7.9)	2.6 (−0.3–5.5)	1.1 (−1.8–4.0)
Often	−1.1 (−6.8–4.5)	−0.3 (−5.9–5.3)	0.8 (−1.7–3.4)	−1.2 (−3.7–1.4)
Occasionally	−2.7 (−8.5–3.0)	−2.2 (−8.0–3.6)	1.8 (−0.7–4.2)	0.1 (−2.4–2.5)
No	0	0	0	0
**Index ^b^**				
0	0	0	0	0
1	−0.2 (−4.2–3.9)	−0.7 (−4.7–3.3)	−2.7 (−4.8–−0.5)	−2.9 (−5.1–−0.7)
2	−1.4 (−6.2–3.4)	−3.7 (−8.4–1.0)	−4.9 (−7.3–−2.5)	−6.8 (−9.2–−4.4)
3	−2.2 (−7.9–3.5)	−4.4 (−10.0–1.2)	−5.0 (−8.0–−2.0)	−6.3 (−9.4–−3.3)
4	−2.4 (−12.1–7.2)	−0.2 (−9.4–9.0)	−7.1 (−11.7–−2.5)	−9.7 (−14.3–−5.1)

^a^ Analyses were adjusted for sex, age and use of supplements containing vitamin D; ^b^ The answers “always” and “often” were combined to “yes” for each sun exposure guideline, while “no” included “occasionally” and “no”.

**Table 3:** Adherence to sun exposure guidelines in relation to odds ratio (OR) of having low vitamin D status ^a^.

	Children (*n* = 569)		Adults (*n* = 2625)	
	Level 1 ^b^ (OR (95% CI))	Level 2 ^c^ (OR (95% CI))	Level 1 ^b^ (OR (95% CI))	Level 2 ^c^ (OR (95% CI))
**Shade**				
Always	3.73 (1.02–13.60)	4.91 (1.47–16.43)	2.10 (1.20–3.69)	1.61 (0.95–2.71)
Often	1.62 (0.72–3.62)	1.14 (0.55–2.37)	2.09 (1.38–3.15)	2.17 (1.50–3.15)
Occasionally	0.88 (0.45–1.72)	0.70 (0.38–1.30)	1.31 (0.88–1.96)	1.16 (0.80–1.69)
No	1	1	1	1
**Sunhat**				
Always	7.26 (1.39–37.81)	-	1.48 (0.82–2.66)	1.22 (0.72–2.06)
Often	1.04 (0.27–3.91)	1.16 (0.37–3.64)	1.22 (0.79–1.87)	0.93 (0.62–1.40)
Occasionally	1.37 (0.58–3.24)	1.85 (0.91–3.75)	0.70 (0.47–1.05)	0.76 (0.53–1.07)
No	1	1	1	1
**Clothing**				
Always	3.59 (1.47–8.74)	2.16 (0.94–4.96)	3.19 (1.98–5.16)	3.09 (2.01–4.74)
Often	1.40 (0.65–3.00)	0.87 (0.43–1.78)	2.31 (1.64–3.25)	1.90 (1.39–2.61)
Occasionally	1.13 (0.58–2.18)	0.73 (0.40–1.33)	1.37 (0.99–1.89)	1.47 (1.10–1.96)
No	1	1	1	1
**Sunscreen**				
Always	1.29 (0.46–3.64)	1.20 (0.45–3.23)	0.70 (0.45–1.07)	0.81 (0.55–1.21)
Often	1.32 (0.53–3.26)	1.41 (0.58–3.41)	0.80 (0.56–1.14)	0.97 (0.70–1.35)
Occasionally	2.36 (0.98–5.70)	1.80 (0.75–4.34)	0.70 (0.49–0.99)	0.86 (0.63–1.19)
No	1	1	1	1

^a^ Analyses were adjusted for sex, age and use of supplements containing vitamin D; ^b^ Level 1: <25 nmol/L either spring or autumn; ^c^ Level 2: <50 nmol/L both spring and autumn.

**Table 4:** Adherence to sun exposure guidelines in relation to odds ratio (OR) of having adequate/high vitamin D status ^a^.

	Children (*n* = 569)		Adults (*n* = 2625)	
	Level 3 ^b^ (OR (95% CI))	Level 4 ^c^ (OR (95% CI))	Level 3 ^b^ (OR (95% CI))	Level 4 ^c^ (OR (95% CI))
**Shade**				
Always	0.20 (0.06–0.68)	0.17 (0.02–1.46)	0.62 (0.37–1.05)	0.64 (0.43–0.93)
Often	0.88 (0.42–1.83)	0.99 (0.54–1.81)	0.46 (0.32–0.67)	0.55 (0.42–0.72)
Occasionally	1.43 (0.77–2.66)	1.07 (0.65–1.78)	0.86 (0.59–1.24)	0.78 (0.60–1.01)
No	1	1	1	1
**Sunhat**				
Always	-	0.75 (0.23–2.40)	0.82 (0.48–1.38)	0.70 (0.46–1.05)
Often	0.86 (0.27–2.68)	0.67 (0.32–1.39)	1.08 (0.71–1.62)	1.07 (0.79–1.46)
Occasionally	0.54 (0.27–1.09)	1.16 (0.69–1.95)	1.32 (0.94–1.87)	1.19 (0.93–1.51)
No	1	1	1	1
**Clothing**				
Always	0.46 (0.20–1.07)	0.81 (0.37–1.76)	0.32 (0.21–0.50)	0.49 (0.33–0.72)
Often	1.14 (0.56–2.34)	0.58 (0.33–1.02)	0.53 (0.38–0.72)	0.53 (0.42–0.68)
Occasionally	1.37 (0.75–2.50)	1.11 (0.69–1.80)	0.68 (0.51–0.91)	0.71 (0.58–0.88)
No	1	1	1	1
**Sunscreen**				
Always	0.83 (0.31–2.24)	1.74 (0.77–3.94)	1.23 (0.83–1.82)	1.68 (1.25–2.25)
Often	0.71 (0.29–1.72)	1.24 (0.57–2.71)	1.03 (0.74–1.43)	1.38 (1.06–1.79)
Occasionally	0.55 (0.23–1.33)	1.16 (0.52–2.60)	1.16 (0.84–1.60)	1.27 (0.99–1.63)
No	1	1	1	1

^a^ Analyses were adjusted for sex, age and use of supplements containing vitamin D; ^b^ Level 3: ≥50 nmol/L either spring or autumn; ^c^ Level 4: ≥50 nmol/L both spring and autumn.

**Table 5:** Vitamin D concentrations among children (2–15 years) in relation to sun exposure guidelines in the Day care/kindergarten/after school club ^a^.

	Spring (Slope (95% CI))	Autumn (Slope (95% CI))
Yes	−0.18 (−5.69–5.32)	−1.89 (−7.15–3.36)
Unsure	−0.090 (−5.32–5.14)	−3.05 (−8.01–1.90)
No	0	0

^a^ Analyses were adjusted for sex, age and use of supplements containing vitamin D.

## Supplementary Materials

The following are available online at http://www.mdpi.com/2072-6643/8/5/266/s1, Table S1: Combined effect of adherence to sun advice and time spent outside: Effect on vitamin D (nmol/L) of adherence to the four pieces of sun advice as well as the combined index in relation to total time spent outside (per hour). The estimates show the effect on vitamin D (nmol/L) per each extra hour per day spent outside in combination with sun advice and the combined index.

## References

[1] Holick M.F. (2007). Vitamin D deficiency. N. Engl. J. Med..

[2] Oliveria S.A., Saraiya M., Geller A.C., Heneghan M.K., Jorgensen C. (2006). Sun exposure and risk of melanoma. Arch. Dis. Child..

[3] Engholm G., Ferlay J., Christensen N., Kejs A.M.T., Johannesen T.B., Khan S., Leinonen M.K., Milter M.C., Ólafsdóttir E., Petersen T. Nordcan: Cancer Incidence, Mortality, Prevalence and Survival in the Nordic Countries, Version 7.2 (16.12.2015), Association of the Nordic Cancer Registries, Danish Cancer Society. http://www.ancr.nu.

[4] Køster B., Thorgaard C., Philip A., Clemmensen I.H. (2010). Prevalence of sunburn and sun-related behaviour in the Danish population: A cross-sectional study. Scand. J. Public Health.

[5] Danish Sun Safety Campaign. https://www.cancer.dk/forebyg/skru-ned-for-solen/english/.

[6] Cancer Research UK Ways to Enjoy the Sun Safely. http://www.cancerresearchuk.org/about-cancer/causes-of-cancer/sun-uv-and-cancer/ways-to-enjoy-the-sun-safely.

[7] Cancer Council Australia Preventing Skin Cancer. http://www.cancer.org.au/preventing-cancer/sun-protection/preventing-skin-cancer/.

[8] American Academy of Dermatology Prevent Skin Cancer. https://www.aad.org/public/spot-skin-cancer/learn-about-skin-cancer/prevent.

[9] World Health Organization Sun Protection. http://www.who.int/uv/sun_protection/en/.

[10] Lips P., van Schoor N.M. (2011). The effect of vitamin D on bone and osteoporosis. Best Pract. Res. Clin. Endocrinol. Metab..

[11] Jacobs E.T., Kohler L.N., Kunihiro A.G., Jurutka P.W. (2016). Vitamin D and Colorectal, Breast, and Prostate Cancers: A Review of the Epidemiological Evidence. J. Cancer.

[12] Mpandzou G., Ait Ben Haddou E., Regragui W., Benomar A., Yahyaoui M. (2016). Vitamin D deficiency and its role in neurological conditions: A review. Rev. Neurol..

[13] Papandreou D., Hamid Z.T. (2015). The Role of Vitamin D in Diabetes and Cardiovascular Disease: An Updated Review of the Literature. Dis. Markers.

[14] Durup D., Jorgensen H.L., Christensen J., Schwarz P., Heegaard A.M., Lind B. (2012). A reverse J-shaped association of all-cause mortality with serum 25-hydroxyvitamin D in general practice: The CopD study. J. Clin. Endocrinol. Metab..

[15] Durup D., Jorgensen H.L., Christensen J., Tjønneland A., Olsen A., Halkjær J., Lind B., Heegaard A.M., Schwarz P. (2015). A Reverse J-Shaped Association between Serum 25-Hydroxyvitamin D and Cardiovascular Disease Mortality: The CopD Study. J. Clin. Endocrinol. Metab..

[16] Tuohimaa P., Tenkanen L., Ahonen M., Lumme S., Jellum E., Hallmans G., Stattin P., Harvei S., Hakulinen T., Luostarinen T. (2004). Both high and low levels of blood vitamin D are associated with a higher prostate cancer risk: A longitudinal, nested case-control study in the Nordic countries. Int. J. Cancer.

[17] Cashman K.D. (2007). Vitamin D in childhood and adolescence. Postgrad. Med. J..

[18] Webb A.R., Engelsen O. (2006). Calculated ultraviolet exposure levels for a healthy vitamin D status. Photochem. Photobiol..

[19] Pedersen C.B. (2011). The Danish Civil Registration System. Scand. J. Public Health.

[20] Danish Health Authority. https://sundhedsstyrelsen.dk/da/sundhed-og-livsstil/ernaering/d-vitamin/forebyggende-d-vitamintilskud.

[21] Bjorn Jensen C., Thorne-Lyman A.L., Vadgaard Hansen L., Strom M., Odgaard Nielsen N., Cohen A., Olsen S.F. (2013). Development and validation of a vitamin D status prediction model in Danish pregnant women: A study of the Danish National Birth Cohort. PLoS ONE.

[22] Tjonneland A., Olsen A., Boll K., Stripp C., Christensen J., Engholm G., Overvad K. (2007). Study design, exposure variables, and socioeconomic determinants of participation in Diet, Cancer and Health: A population-based prospective cohort study of 57,053 men and women in Denmark. Scand. J. Public Health.

[23] Danish Sun Safety Campaign A Survey of the Sun Habits of the Danish Population in Denmark during the Summer of 2010. https://www.cancer.dk/forebyg/skru-ned-for-solen/forskning-og-evaluering/rapporter/.

[24] Madsen K.H., Rasmussen L.B., Andersen R., Mølgaard C., Jakobsen J., Bjerrum P.J., Andersen E.W., Mejborn H., Tetens I. (2013). Randomized controlled trial of the effects of vitamin D-fortified milk and bread on serum 25-hydroxyvitamin D concentrations in families in Denmark during winter: The VitmaD study. Am. J. Clin. Nutr..

[25] Centers for Disease Control and Prevention National Center for Health Statistics. Z-score Data Files. http://www.cdc.gov/growthcharts/zscore.htm.

[26] World Health Organization BMI-for-Age (5–19 Years). http://www.who.int/growthref/who2007_bmi_for_age/en/.

[27] Ross A.C., Taylor C.L., Yaktine A.L., Del Valle H.B. (2011). Dietary Reference Intakes for Calcium and Vitamin D.

[28] Wulf H.C., Eriksen P. (2010). UV index and its implications. Ugeskr. Laeger.

[29] Linos E., Keiser E., Kanzler M., Sainani K.L., Lee W., Vittinghoff E., Chren M.M., Tang J.Y. (2012). Sun protective behaviors and vitamin D levels in the US population: NHANES 2003–2006. Cancer Causes Control.

[30] Matsuoka L.Y., Ide L., Wortsman J., MacLaughlin J.A., Holick M.F. (1987). Sunscreens suppress cutaneous vitamin D3 synthesis. J. Clin. Endocrinol. Metab..

[31] Autier P., Boniol M., Dore J.F. (2007). Sunscreen use and increased duration of intentional sun exposure: Still a burning issue. Int. J. Cancer.

[32] Zerwekh J.E. (2008). Blood biomarkers of vitamin D status. Am. J. Clin. Nutr..

[33] Cashman K.D., Dowling K.G., Skrabakova Z., Kiely M., Lamberg-Allardt C., Durazo-Arvizu R.A., Sempos C.T., Koskinen S., Lundqvist A., Sundvall J. (2015). Standardizing serum 25-hydroxyvitamin D data from four Nordic population samples using the vitamin D Standardization Program Protocols: Shedding new light on vitamin D status in Nordic individuals. Scand. J. Clin. Lab. Investig..

[34] Glanz K., Gies P., O’Riordan D.L., Elliott T., Nehl E., McCarty F., Davis E. (2010). Validity of self-reported solar UVR exposure compared with objectively measured UVR exposure. Cancer Epidemiol. Biomark. Prev..

[35] Thieden E. (2008). Sun exposure behaviour among subgroups of the Danish population. Based on personal electronic UVR dosimetry and corresponding exposure diaries. Dan. Med. Bull..

[36] Williams S., Malatesta K., Norris K. (2009). Vitamin D and chronic kidney disease. Ethn. Dis..

