# Supplementary material for: Sun Exposure Guidelines and Serum Vitamin D Status in Denmark: The StatusD Study
Source: Nutrients. 2016 May 5;8(5):266. doi: 10.3390/nu8050266 (PMC4882679; doi:10.3390/nu8050266)
Supplement: Supplementary file 1 [file nutrients-08-00266-s001.pdf]

Supplementary Materials: Sun Exposure Guidelines and Serum Vitamin D Status in Denmark: The StatusD Study

Louise Hansen, Anne Tjønneland, Brian Køster, Christine Brot, Rikke Andersen, Marika Lundqvist, Jane Christensen and Anja Olsen

Table S1. Combined effect of adherence to sun advice and time spent outside: Effect on vitamin D (nmol/L) of adherence to the four pieces of sun advice as well as the combined index in relation to total time spent outside (per hour). The estimates show the effect on vitamin D (nmol/L) per each extra hour per day spent outside in combination with sun advice and the combined index [a,b].

	**Children (*n* = 569)**		**Adults (*n* = 2625)**	
	**Spring**	**Autumn**	**Spring**	**Autumn**
**Adherence to sun exposure guideline on shade**				
Always	0.28 (−1.74–2.3) *	−0.80 (−2.53–0.95) *	0.17 (−0.13–0.46)	0.078 (−0.22–0.38)
Often	−0.09 (−0.59–0.42) *	−0.17 (−0.62–0.28) *	0.063 (−0.10–0.23)	0.068 (−0.01–0.24)
Occasionally	0.41 (0.09–0.73) *	0.64 (0.33–0.95) *	0.25 (0.11–0.39)	0.49 (0.35–0.63)
No	−0.16 (−0.76–0.44) *	0.079 (−0.51–0.67) *	0.53 (0.30–0.75)	0.64 (0.42–0.87)
**Adherence to sun exposure guideline on sunhat**				
Always	0.21 (−0.96–1.39)	0.75 (−0.42–1.92)	0.12 (−0.20–0.45)	0.093 (−0.24–0.43)
Often	0.08 (−0.60–0.76)	0.37 (−0.30–1.04)	0.09 (−0.14–0.32)	0.28 (0.053–0.51)
Occasionally	0.42 (−0.16–1.00)	0.44 (−0.083–0.96)	0.37 (0.16–0.57)	0.51 (0.30–0.72)
No	0.25 (−0.07–0.56)	0.36 (0.056–0.66)	0.24 (0.11–0.37)	0.37 (0.24–0.50)
**Adherence to sun exposure guideline on protective clothing**				
Always	−0.07 (−0.80–0.66)	−0.04 (−0.77–0.69)	−0.22 (−0.57–0.12)	−0.23 (−0.58–0.13)
Often	−0.27 (−0.87–0.34)	0.23 (−0.24–0.69)	0.055 (−0.15–0.26)	0.053 (−0.15–0.25)
Occasionally	0.26 (−0.14–0.66)	0.30 (−0.10–0.69)	0.12 (−0.04–0.27)	0.27 (0.11–0.43)
No	0.41 (0.03–0.78)	0.61 (0.23–0.99)	0.42 (0.27–0.56)	0.60 (0.46–0.74)
**Adherence to sun exposure guideline on sunscreen**				
Always	0.34 (−0.02–0.71)	0.51 (0.18–0.84)	0.35 (0.14–0.55)	0.50 (0.30–0.71)
Often	0.21 (−0.25–0.67)	0.22 (−0.23–0.67)	0.27 (0.09–0.46)	0.30 (0.11–0.49)
Occasionally	0.12 (−0.34–0.57)	0.22 (−0.24–0.67)	0.27 (0.11–0.43)	0.35 (0.18–0.51)
No	0.19 (−1.02–1.40)	1.11 (−0.22–2.43)	0.087 (−0.08–0.26)	0.35 (0.18–0.52)
**Combined sun exposure guideline index [c]**				
0	0.16 (−0.33–0.66)	0.47 (−0.032–0.97)	0.34 (0.18–0.49)	0.50 (0.35–0.66)
1	0.42 (0.05–0.78)	0.60 (0.25–0.96)	0.22 (0.059–0.39)	0.51 (0.34–0.68)
2	0.22 (−0.29–0.75)	0.084 (−0.39–0.56)	0.094 (−0.098–0.29)	0.11 (−0.08–0.30)
3	−0.20 (−0.98–0.57)	−0.066 (−0.67–0.54)	0.18 (−0.098–0.29)	0.14 (−0.01–0.38)
4	−0.34 (−1.25–0.57)	0.39 (−0.51–1.29)	−0.07 (−0.51–0.38)	−0.25 (−0.79–0.20)

[a] Analyses were adjusted for sex, age and use of supplements containing vitamin D and have "time spent outside" as an interaction variable; [b] p for interaction < 0.05 for models in spring and autumn for sun exposure guideline on shade among children only (shown with an asterisk); [c] The answers "always" and "often" were combined to "yes" for each sun exposure guideline, while "no" included "occasionally" and "no".
